# ACHR.cu: GPU-accelerated sampling of metabolic networks

**DOI:** 10.21105/joss.01363

**Published:** 2019-11-27

**Authors:** Marouen Ben Guebila

**Affiliations:** 1 Luxembourg Centre for Systems Biomedicine, University of Luxembourg, Esch-sur-Alzette, Luxembourg.

## Introduction

The *in silico* modeling of biological organisms consists of the mathematical representation of key functions of a biological system and the study of its behavior in different conditions and environments. It serves as a tool to support wet lab experiments and to generate hypotheses about the functioning of the subsystems. Among the many biological products, metabolism is the most amenable to modeling because it is directly related to key biological functions and processes. Moreover, public data resources of several metabolites and their abundances have been developing rapidly in recent years thereby enabling applications in many areas. In biotechnology, the metabolic modeling of ethanol-producing bacteria allows finding key interventions, such as substrate optimization, that would increase the yield in the bioreactor and improve its efficiency (Mahadevan, Burgard, Famili, Dien, & Schilling, 2005).

Recently, high-throughput technologies allowed to generate a large amount of biological data that enabled complex modeling of biological systems. As models expand in size, the tools used for their analysis have to be appropriately scaled to include the use of parallel software.

A tool of choice for the analysis of metabolic models is the sampling of the space of their possible phenotypes. Instead of considering one specific biological function of interest, sampling is an unbiased tool for metabolic modeling that explores all the space of possible metabolic phenotypes. For large models, sampling becomes expensive both in time and computational resources. To make sampling accessible in the modeler’s toolbox, we present ACHR.cu which is a fast Graphical Processing Unit (GPU) implementation of the sampling algorithm Artificial Centering Hit-and-Run (ACHR) (Kaufman & Smith, 1998) using the parallel computing platform CUDA (Nickolls, Buck, & Garland, 2008).

## Results

Metabolic models are networks of *m* metabolites involved in *n* reactions. Briefly, they are formulated as linear programs (O’Brien, Monk, & Palsson, 2015) using the stoichiometric matrix *S*_(*m,n*)_. A central hypothesis in metabolic modeling is the steady-state assumption, meaning that generating fluxes are equal to degrading fluxes and the rate of change of metabolite concentrations is null. Mathematically, the steady-state assumption translates to constraining the solution space to the null space of the stoichiometric matrix *S*_(*m,n*)_.

The solution to the linear program allows finding flux values in the network that achieve the objective function of interest. Particularly, ACHR allows obtaining a distribution of possible fluxes for each reaction.

The sampling of the solution space of metabolic models is a two-step process:

### Generation of warmup points

The first step of sampling the solution space of metabolic models involves the generation of warmup points that are solutions to the metabolic model’s linear program. The sampling chain starts from those solutions to explore the solution space.

The generation of *p* ≥ 2*n* warmup points corresponds to flux variability analysis (FVA) (Mahadevan & Schilling, 2003) solutions for the first 2*n* points, with *n* the number of reactions in the network and the objective function is to minimize and maximize each reaction in the model (hence 2*n*). For the remaining *p* – 2*n* points, they correspond to solutions generated using a random objective vector *c*_(*n*.1)_ in the linear program.

The generation of warmup points is a time-consuming process and requires the use of more than one core in parallel. The distribution of the points to generate among the *nc* cores of the computer is often performed through static balancing with each core getting *p/nc* points to generate. Nevertheless, the formulation of the problem induces a significant imbalance in the distribution of work, meaning that the workers will not converge at the same time thereby slowing down the overall process. We showed previously that FVA is imbalanced, especially with metabolism-expression models (Guebila, 2018). The generation of warmup points through random *c* vectors of objective coefficients is yet another factor to favor ill-conditioned problems and the imbalance between the parallel workers.

To address the imbalance between the workers, dynamic loading balancing as implemented through the OpenMP parallel library in C (Dagum & Menon, 1998) allows assigning fewer points to workers that required more time to solve previous chunks of reactions. In the end, the workers converge at the same time.

Given this background, the generation of 30,000 warmup points using an OpenMP dynamically load balanced implementation (CreateWarmupVF) (Guebila, 2018) and the MATLAB version (CreateWarmupMATLAB) were compared on three metabolic models, i.e., E. coli core (Orth, Palsson, & Fleming, 2010), P. putida (Nogales, Palsson, & Thiele, 2008), and Recon2 (Thiele et al., 2013). The speedup achieved by CreateWarmupVF over CreateWarmupMATLAB was substantial (up to 50 fold) (Guebila, 2018) and showed the power of dynamic load balancing in ill-conditioned parallel problems. Using the generated warmup points, the uniform sampling process can start to explore the solution space.

### Sampling of the solution space

Second, the sampling of the solution space of metabolic models involves the generation of sampling chains starting from the warmup points. The sampling in MATLAB was performed using the ACHR serial function using one sampling chain, and each point was saved after 1000 steps. The GPU parallel version (ACHR.cu) creates one chain for each point executed by one thread in the GPU. Moreover, each thread can call additional threads to perform large matrix operations using the grid nesting and dynamic parallelism capabilities of the new NVIDIA cards (sm_35 and higher). When compared to the CPU, the speedup with the GPU is quite important as reported in table 1. It is noteworthy that even for a single core, the CPU is multithreaded especially with optimized MATLAB base functions such as min and max, and that despite a large number of cores in the GPU, they are slow (0.7 GHz) in comparison to CPU (3.5 GHz).

The implementation of null space computation to constrain the metabolic model was a significant determinant in the final runtime, and the fastest implementation was reported (Table 1). Particularly, there was a tradeoff between memory usage and access, and the computation time when either QR or Singular Value Decomposition (SVD) was used.

While computing the SVD of the *S* matrix is generally more precise than QR, it is not prone to parallel computation in the GPU which can be even slower than the CPU in some cases. However, computing the null space through QR decomposition is faster but less precise and consumes more memory as it takes all the dimensions of the matrix in contrast to SVD that removes columns below a given precision of the singular values.

Finally, ACHR.cu was developed as a high-performance tool for the modeling of metabolic networks using a parallel architecture that segregates the generation of warmup points and the sampling.

## Comparison to existing software

Conceptually, the architecture of the parallel GPU implementation of ACHR.cu is similar to the MATLAB Cobra Toolbox (Heirendt et al., 2019) GpSampler. Another tool, OptGpSampler (Megchelenbrink, Huynen, & Marchiori, 2014) provides up to 40 fold speedup over GpSampler through a i) C implementation and ii) fewer but longer sampling chains launch. Since OptGpSampler performs the generation of the warmup points and the sampling in one process, it is clear from the results of the current work that the speedup achieved with the generation of warmup points is more significant than sampling itself. We decoupled the generation of warmup points from sampling to take advantage of dynamic load balancing with OpenMP. Additionally, in OptGpSampler each worker gets the same amount of points and steps to compute; the problem is statically load balanced by design. In contrast, when the generation of warmup points is performed separately from sampling, the problem can be dynamically balanced and the parallel workers are ensured to converge simultaneously.

Finally, future improvements of this work can consider an MPI/CUDA hybrid to take advantage of the multi-GPU architecture of recent NVIDIA cards like the K80. Additionally, the integration of LP solvers on the GPU (Charlton, Maddock, & Richmond, 2019; Gurung & Ray, 2019; Li, Lv, Hu, & Jiang, 2011) can help the development of end-to-end sampling solutions. Taken together, the parallel architecture of ACHR.cu allows faster sampling of metabolic models over existing tools thereby enabling the unbiased analyses of large-scale systems biology models.

## Figures and Tables

**Table 1:** Runtimes in seconds of ACHR in MATLAB and ACHR.cu for a set of metabolic models starting from 30,000 warmup points. SVD and QR refer to the implementation of the null space computation, *m* and *n* respectively refer to the number of metabolites and reactions in the model.

Model	*m/n*	Points	Steps per point	Intel Xeon (3.5 GHz)	Tesla K40
E. coli core	72/95	1000	1000	42	2 (SVD)
E. coli core	72/95	5000	1000	208	12 (SVD)
E. coli core	72/95	10000	1000	420	24 (SVD)
P. putida	911/1060	1000	1000	103	17 (SVD)
P. putida	911/1060	5000	1000	516	70 (SVD)
P. putida	911/1060	10000	1000	1081	138 (SVD)
Recon2	4036/7324	1000	1000	2815	269 (QR)
Recon2	4036/7324	5000	1000	14014	1110 (QR)
Recon2	4036/7324	10000	1000	28026	2240 (QR)
